# Supplementary material for: Clinical Features of LMNA-Related Cardiomyopathy in 18 Patients and Characterization of Two Novel Variants
Source: J Clin Med. 2021 Oct 29;10(21):5075. doi: 10.3390/jcm10215075 (PMC8584896; doi:10.3390/jcm10215075)
Supplement: Supplementary file 1 [file jcm-10-05075-s001.zip › jcm-1394979-supplementary.pdf]

Supplementary Table 1: Genes studied for Sudden Cardiac Death

Gene	***Phenotype***	***Gene***	***Phenotype***
ABCC9	Atrial fibrillation, familial, 12	*LDB3*	Cardiomyopathy, dilated, 1C, with or without LVNC
	Cardiomyopathy, dilated,		Cardiomyopathy, hypertrophic, 24
			Left ventricular noncompaction 3
			Myopathy, myofibrillar, 4
ACTC1	Atrial septal defect 5	*LMNA*	Cardiomyopathy, dilated, 1°
	Cardiomyopathy, dilated, 1R		Charcot-Marie-Tooth disease
	Cardiomyopathy, hypertrophic, 11		Lipodystrophy, familial partial, type
	Left ventricular noncompaction 4		Mandibuloacral dysplasia
ACTN2	Cardiomyopathy, dilated, 1AA, with or without LVNC	*MYBPC3*	Cardiomyopathy, dilated, 1MM
	Cardiomyopathy, hypertrophic, 23, with or without LVNC		Cardiomyopathy, hypertrophic, 4
			Left ventricular noncompaction 10
AKAP9	Long QT syndrome-11	*MYH6*	Atrial septal defect 3
			Cardiomyopathy, dilated, 1EE
			Cardiomyopathy, hypertrophic, 14
ANK2	Cardiac arrhythmia, ankyrin-B-related	*MYH7*	Cardiomyopathy, dilated, 1S
	Long QT syndrome 4		Cardiomyopathy, hypertrophic, 1
			Left ventricular noncompaction 5
			Myopathy, myosin storage
			Scapuloperoneal syndrome, myopathic type
BAG3	Cardiomyopathy, dilated, 1HH	*MYL2*	Cardiomyopathy, hypertrophic, 10
	Myopathy, myofibrillar, 6		
CACNA1C	Brugada syndrome 3	*MYL3*	Cardiomyopathy, hypertrophic, 8
	Timothy syndrome		
CACNB2	Brugada syndrome 4	*NEXN*	Cardiomyopathy, dilated, 1CC
			Cardiomyopathy, hypertrophic, 20
CALR3	Cardiomyopathy, hypertrophic, 19	*PKD2*	Polycystic kidney disease 2

CASQ2	Ventricular tachycardia, catecholaminergic polymorphic, 2	*PKP2*	Arrhythmogenic right ventricular dysplasia 9
CAV3	Cardiomyopathy, familial hypertrophic	*PLN*	Cardiomyopathy, dilated, 1P
	Creatine phosphokinase, elevated serum		Cardiomyopathy, hypertrophic, 18
	Long QT syndrome 9		
	Muscular dystrophy, limb-girdle, type IC		
	Myopathy, distal, Tateyama type		
	Rippling muscle disease		
CSRP3	Cardiomyopathy, dilated, 1M	*PRKAG2*	Cardiomyopathy, hypertrophic 6
	Cardiomyopathy, hypertrophic, 12		Glycogen storage disease of heart, lethal congenital
			Wolff-Parkinson-White syndrome
DES	Cardiomyopathy, dilated, 1I	*PSEN1*	Acne inversa, familial
	Myopathy, myofibrillar, 1		Alzheimer disease
	Scapuloperoneal syndrome, neurogenic, Kaeser type		Cardiomyopathy, dilated, 1U
			Dementia, frontotempora
			Pick disease
DMD	Becker muscular dystrophy	*PSEN2*	Alzheimer disease-4
	Cardiomyopathy, dilated, 3B		Cardiomyopathy, dilated, 1V
	Duchenne muscular dystrophy		
DSC2	Arrhythmogenic right ventricular dysplasia 11	*RBM20*	Cardiomyopathy, dilated, 1DD
	Arrhythmogenic right ventricular dysplasia 11 with mild palmoplantar keratoderma and woolly hair		
DSG2	Arrhythmogenic right ventricular dysplasia 10	*RYR2*	Arrhythmogenic right ventricular dysplasia 2
	Cardiomyopathy, dilated, 1BB		Ventricular tachycardia, catecholaminergic polymorphic, 1
DSP	Arrhythmogenic right ventricular dysplasia 8	*SCN1B*	Atrial fibrillation, familial, 13

	Cardiomyopathy, dilated, with woolly hair and keratoderma		Brugada syndrome 5
	Dilated cardiomyopathy with woolly hair, keratoderma, and tooth agenesis		Cardiac conduction defect, nonspecific
	Skin fragility-woolly hair syndrome		Epilepsy, generalized, with febrile seizures plus, type 1
DTNA	Left ventricular noncompaction 1, with or without congenital heart defects	*SCN3B*	Atrial fibrillation, familial, 16
			Brugada syndrome 7
EYA4	Cardiomyopathy, dilated, 1J	*SCN4B*	Atrial fibrillation, familial, 17
	Deafness, autosomal dominant 10		Long QT syndrome-10
FKTN	Cardiomyopathy, dilated, 1X	*SCN5A*	Brugada syndrome 1
	Muscular dystrophy-dystroglycanopathy		Cardiomyopathy, dilated, 1E
			Heart block
			Long QT syndrome-3
GATAD1	Cardiomyopathy, dilated, 2B	*SDHA*	Cardiomyopathy, dilated, 1GG
			Leigh syndrome
			Mitochondrial respiratory chain complex II deficiency
			Paragangliomas 5
GLA	Fabry disease	*SGCD*	Cardiomyopathy, dilated, 1L
	Fabry disease, cardiac variant		Muscular dystrophy, limb-girdle, type 2F
GPD1L	Brugada syndrome 2	*SNTA1*	Long QT syndrome 12
HCN4	Brugada syndrome 8	*TAZ*	Barth syndrome
	Sick sinus syndrome		
JPH2	Cardiomyopathy, hypertrophic, 17	*TCAP*	Cardiomyopathy, hypertrophic, 25
			Muscular dystrophy, limb-girdle, type 2G
JUP	Arrhythmogenic right ventricular dysplasia 12	*TGFB3*	Arrhythmogenic right ventricular dysplasia 1
	Naxos disease		Loeys-Dietz syndrome 5
KCNA5	Atrial fibrillation, familial, 7	*TMEM43*	Arrhythmogenic right ventricular dysplasia

			Emery-Dreifuss muscular dystrophy 7
KCNE1	Long QT syndrome 5	*TMPO*	
	Jervell and Lange-Nielsen syndrome 2		
KCNE2	Atrial fibrillation, familial, 4	*TNNC1*	Cardiomyopathy, dilated, 1Z
	Long QT syndrome 6		Cardiomyopathy, hypertrophic, 13
KCNE3	Brugada syndrome 6	*TNNI3*	Cardiomyopathy, dilated,
			Cardiomyopathy, hypertrophic, 7
			Cardiomyopathy, hypertrophic, 7
KCNH2	Long QT syndrome 2	*TNNT2*	Cardiomyopathy, dilated, 1D
	Short QT syndrome 1		Cardiomyopathy, familial restrictive, 3
			Cardiomyopathy, hypertrophic, 2
			Left ventricular noncompaction 6
KCNJ2	Andersen syndrome	*TPM1*	Cardiomyopathy, dilated, 1Y
	Atrial fibrillation, familial, 9		Cardiomyopathy, hypertrophic, 3
	Short QT syndrome 3		Left ventricular noncompaction 9
KCNJ5	Hyperaldosteronism, familial, type III	*TTN*	Cardiomyopathy, dilated, 1G
	Long QT syndrome		Cardiomyopathy, familial hypertrophic, 9
			Muscular dystrophy, limb-girdle, type 2J
			Myopathy, proximal, with early respiratory muscle involvement
			Salih myopathy
			Tibial muscular dystrophy, tardive
KCNQ1	Atrial fibrillation, familial, 3	*TTR*	Amyloidosis, hereditary, transthyretin-related
	Jervell and Lange-Nielsen syndrome		Carpal tunnel syndrome, familial
	Long QT syndrome 1		
	Short QT syndrome 2		
LAMP2	Danon disease	*VCL*	Cardiomyopathy, dilated, 1W
			Cardiomyopathy, hypertrophic, 15
